# Exploring the Mechanisms Linking Transformational Leadership, Perceived Organizational Support, Creativity, and Performance in Hospitality: The Mediating Role of Affective Organizational Commitment

**DOI:** 10.3390/bs12100406

**Published:** 2022-10-21

**Authors:** Chung-Jen Wang

**Affiliations:** Department of Hotel and Restaurant Management, National Pingtung University of Science and Technology, Shuefu Road, Neipu, Pingtung 912301, Taiwan; wchungzen@gmail.com

**Keywords:** affective organizational commitment, creativity, perceived organizational support, performance, transformational leadership, human health, sustainable growth, hospitality

## Abstract

Integrating leadership and creativity theories, this study proposed and examined a model linking transformational leadership, perceived organizational support, affective organizational commitment, creativity, and performance. Structural equation modeling was thus performed using 362 employee–supervisor dyads from the international tourist hotels in Taiwan. The results indicated that both transformational leadership and perceived organizational support were significantly and positively related to employee affective organizational commitment, creativity, and performance, while affective organizational commitment had significantly positive influences on creativity and performance. Support was also found for the significant and positive mediating relationships between affective organizational commitment and the influences of both transformational leadership and perceived organizational support on creativity and performance. The theoretical and practical implications of these findings for hospitality are also discussed.

## 1. Introduction

During the COVID-19 pandemic period, all the global sectors have faced the unprecedented crisis regarding maintaining a thriving business. However, the hospitality and tourism industry experienced major changes in leadership for its operating environment. As leaders are people who inspire or guide others, their styles of leadership are expected to promote collaboration, control information, and clarify decision making for the industry’s recovery. Among different leadership styles, transformational leadership has been one of the most successful management tools for inspiring people to make positive changes and achieve unexpected results [1,2,3,4,5]. Accordingly, in the highly competitive nature of the hospitality and tourism industry, transformational leadership has been widely used to measure leadership effectiveness. Bass and Steidlmeier defined transformational leadership [6] using four dimensions: inspirational motivation, idealized influence, individualized consideration, and intellectual stimulation for encouraging followers to carry out a compelling vision through simple methods. This is an idealized role model for followers that provides vision, gives personal attention, and inspires followers by reframing problems and promoting intelligence using novel approaches to solve problems. Therefore, transformational leaders focus on being an idealized role model, giving employees useful feedback, encouraging followers through high self-reinforcement, motivating them to adopt useful solutions, and promoting them to make further contributions [6,7]. In other words, by affecting followers’ basic values, beliefs, and attitudes, leaders’ transformational behaviors can improve employees’ intrinsic motivation to achieve organizational goals with novel ideas, thus enhancing individual and organizational performance [8,9,10,11,12,13,14]. In contrast, Eisenberger et al. developed the concept of perceived organizational support [15] to assess employees’ perception of the organization’s commitment to them. Perceived organizational support is based on favorable treatment from the organization, such as adequate job training, attractive work conditions, and health care benefits [15,16]. With high levels of perceived organizational support, employees believe that the organization cares about them and values their contributions; if firms are able to build a reciprocal relationship with employees, this scenario tends to promote their creative behavior and performance at work [16,17,18]. Accordingly, this study first aimed to synthesize both transformational leadership and employee perceptions of organizational support in an integrated model and to explore their influences on creativity and performance.

Furthermore, many studies have presented empirical evidence that positive relationships with leaders and organizations can lead employees to feel greater support for their goals, as expressed through factors such as affective organizational commitment [15,19,20,21,22]. As affective organizational commitment represents an employee’s affective attachment, identification, and involvement in his or her organization [23], this positive attitude at work has been recognized as an aspect supporting the organization [24,25]. Most importantly, this affective organizational commitment will enhance employees’ feelings of confidence, competence, and self-determination and thus foster other positive outcomes with regard to individual creativity and work performance [26,27,28,29]. To better understand the findings of prior studies, it will be helpful to develop a mechanism that can explain how transformational leadership and perceived organizational support influence affective organizational commitment, thus leading to creativity and performance. Consequently, we proposed that affective organizational commitment is a vital mediator linking transformational leadership, perceived organizational support, creativity, and performance.

Based on 362 employee–supervisor dyadic data from the international tourist hotels in Taiwan, we examine a theoretical model in the context of hospitality to demonstrate the direct relationship between transformational leadership and perceived organizational support with regard to employee creativity and performance, as well as the indirect relations between transformational leadership and perceived organizational support with regard to subordinate creativity and performance through the mediator of affective organizational commitment. Our research results broaden those of previous studies by integrating transformational leadership, perceived organizational support, affective organizational commitment, creative behavior, and work performance in a corporate setting. Most important of all, our study extends the findings of prior studies, which were mostly carried out in Western contexts, as it was conducted on the basis of data from Taiwanese employees.

Overall, this study aims to synthesize theories by theoretically and empirically linking the concepts of transformational leadership, social exchange, and creativity in hospitality, as well as to provide a broader understanding of their relationships with employee creativity and performance at work.

## 2. Theory and Hypotheses

**Figure 1:**
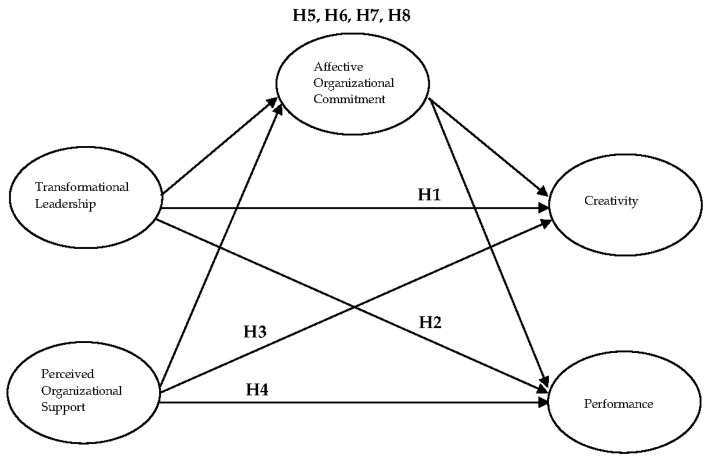
Proposed model.

Integrating leadership and creativity theories, this study proposed and examined a model linking transformational leadership [6], perceived organizational support [15], affective organizational commitment [23], creativity, and performance [8]. In this section, we first investigate the effects of transformational leadership on employee creativity and performance. We next examine how perceived organizational support influences employee creativity and performance. Finally, we explore the mediating role of affective organizational commitment with regard to the links between transformational leadership, perceived organizational support, creativity, and performance. Especially in the competitive business environment of the post COVID-19 era, employees’ high commitment to their hotels can thus contribute to hotel profitability. Figure 1 shows the proposed theoretical model.

### 2.1. Relationships between Transformational Leadership and Creativity and Performance

Leadership is one of the most important contextual factors at work with regard to employee attitudes and behaviors [30]. Among various styles of leadership, increasing attention has been paid to the influences of leaders with transformational leadership qualities [6,7,9,26,31]. Bass and Steidlmeier defined transformational leadership [6] using four dimensions: inspirational motivation (i.e., cooperatively encouraging followers to accomplish a compelling vision), idealized influence or charisma (i.e., being an idealized role model for followers and positively affecting their behaviors and perspectives), individualized consideration (i.e., supporting and understanding different followers’ requirements and development), and intellectual stimulation (i.e., inspiring and stimulating followers by reframing problems, raising the intellectual curiosity of employees, and adopting novel approaches to solve problems). In addition, creativity is defined as generating ideas that are useful and novel for products or processes [8,32,33]. In other words, creativity can be regarded as offering acceptable and adoptable solutions in relative domains [34,35], and it has been widely recognized as a vital ingredient of organizational effectiveness [18,30]. According to Amabile’s [8] componential theory of creativity, the individual factors influencing employee creativity include expertise, creativity skills, and intrinsic motivation. Among these, intrinsic motivation is based on an employee’s sense of enjoyment, curiosity, and involvement regarding his or her work, and individuals with high intrinsic motivation have a high tendency to obtain and apply the new knowledge and skills required for their work [8,36]. Following these perspectives, transformational leaders inspire followers’ intrinsic motivation by self-reinforcement [6,7] and thus help employees achieve organizational goals with novel ideas, as well as high creative performance [9,31]. Particularly in the field of hospitality, hotels need several creative employees to provide knowledge-intensive services; thus, transformational leadership is regarded as the most important environmental feature for individual creativity [1,2,4,37,38]. A number of previous studies have proven this point. For example, using data from 163 employees, Gumusluoglu and Ilsev [11] found that transformational leadership has a positive relationship with employee creativity. Gong et al. [9] also revealed that transformational leadership and learning orientation both have positive relationships with creativity. Similarly, Wang et al. [4] proposed that transformational leadership can positively affect employees’ creative self-efficacy and creativity based on a sample of 395 leader–subordinate dyads from international tourist hotels. Accordingly, this study argues that transformational leadership influences creativity, and it proposes the following hypothesis:

**Hypothesis** **1.** *Transformational leadership is positively related to employee creativity.*

In addition, one of the core values of transformational leadership is emphasizing the development of employees [39,40]. Therefore, transformational leaders concentrate on providing employees with useful feedback, being an idealized role model, inspiring followers with self-reinforcement, motivating them to find useful solutions, and encouraging them to make remarkable contributions [6,7]. In this vein, leaders exhibiting transformational leadership can help to develop subordinates’ potential abilities, thus leading to their improved work performance. More specifically, with the care and support from their leaders, employees tend to make additional commitments at work and express interest in other job responsibilities [9,40]. Accordingly, transformational leaders improve the relevance and importance of the subordinates’ work; therefore, these employees will thus have an increased willingness to take extra efforts to achieve work performance [2,3,41]. For example, Wang et al. [41] stated that transformational leadership can positively influence performance using data from 162 supervisor–employee dyads. Wang, Oh, Courtright, and Colbert [42] also reported that transformational leadership has a positive effect on performance, based on a meta-analytic review of 117 independent samples. Moreover, Dvir et al. [40] carried out a longitudinal experiment and proposed that transformational leadership has a positive influence on direct subordinates’ work performance. As a number of prior studies support the existence of positive relationships between leaders’ transformational leadership and followers’ performance, we thus propose the following hypotheses:

**Hypothesis** **2.** *Transformational leadership is positively related to employee performance.*

### 2.2. Relationships of Perceived Organizational Support with Creativity and Performance

Blau [43] proposed a social exchange theory and suggested that high-quality exchange relationships are symbols of mutual trust and support between the parties concerned. Following this perspective, Eisenberger et al. [15] conceptualized the definition of perceived organizational support as employees’ beliefs that their organizations care about them and value their contributions; they then suggested that perceived organizational support can be associated with employee work attitude and behavior, such as diligence, commitment, and creative performance. Therefore, as talented individuals with expertise and creative thinking abilities are required to successfully carry out their assigned tasks, organizations should stress their support for employees’ creative behavior and performance by using encouragement, rewards, respect, and other forms of recognition [27]. In other words, when employees feel they are valued by their organizations, the failure to implement these novel ideas can be reduced while effectively achieving high performance. Considering that changing the existing system with an innovative approach is difficult, employees will only express their creativity in organizations if they recognize that management systems exhibit allowance, welcome, and acceptance of their meaningful efforts [27,32]. For instance, Francese [44] proposed that organizations focus on support, and adaption can encourage employees’ autonomy and creativity, based on a sample of 38 hotels. Based on a research model with 461 samples, Pundt, Martins, and Nerdinger [45] revealed that employees’ perceived organizational support can influence their innovative behavior. In addition, using 456 leader–subordinate dyads, De Stobbeleir, Ashford, and Buyens [46] found that employees’ perceived organizational support can influence their feedback-seeking and stimulate creative outcomes. Therefore, we propose the following hypothesis:

**Hypothesis** **3.** *Perceived organizational support is positively related to employee creativity.*

Employees’ levels of perceived organizational support reflect their perceptions of care and emphasis from the organization [15,16,47]. If employees have high perceived organizational support, they will feel respected and recognized at work [47]. Thus, they tend to have extra obligations to stay with the organization and continue to voluntarily contribute their efforts. Based on these reciprocal relationships, employees in an organization with high perceived organizational support can reduce their turnover intention, increase career satisfaction, and ultimately promote work performance [48]. Especially given the labor-intensive nature of the hotel business, employees’ perception of organizational support is the key driver of their enhanced performance, enabling them to provide better processes, products, and strategies at work [48,49,50]. For example, based on a study of 245 employees, Rich et al. [51] revealed that perceived organizational support positively influences job performance and organizational citizenship behavior. Based on data obtained from hotel employees, Karatepe [48] indicated that perceived organizational support has positive effects on performance and service recovery via career satisfaction. In addition, Rhoades and Eisenberger [52] showed that employees’ perceptions of organizational support are positively related to their affective commitment and work performance, based on a meta-analysis of their research. Based on these studies, we thus propose the following hypothesis:

**Hypothesis** **4.** *Perceived organizational support is positively related to employee performance.*

### 2.3. Mediating Role of Affective Organizational Commitment

Considerable efforts have been made to uncover the antecedences and consequences of employee organizational commitment [19,53,54]. Allen and Meyer [23] conceptualized organizational commitment as a three-component model including affective, continuance, and normative commitment. Among these three components, affective organizational commitment represents employees’ affective attachment, individual identification, and involvement in the organization. Through transformational leadership, leaders can increase employee affective organizational commitment and their feelings of belonging to the organization by showing support and consideration, serving as role models to improve the capabilities of their followers to generate new ideas and solve problems [19,54]. As for the hospitality industry, these committed employees can be the most valuable asset for the hotels, thus helping to meet the expectations of their customers [55,56,57,58,59]. Preliminary evidence suggests that transformational leadership is positively related to subordinates’ affective organizational commitment. For instance, Barling et al. [19] found that transformational leadership has positive and significant relationships with employee organizational commitment and work performance. Koh et al. [60] reported that transformational leadership has a significant predictive power with regard to organizational commitment, job satisfaction, and organizational citizenship behavior. Similarly, Cole and Bedeian [54] revealed that, based on a sample of 828 workers, transformational leadership is a contextual factor that affects the level of emotional exhaustion and work commitment seen in employees.

Moreover, research has demonstrated that employee creativity is enhanced by a person’s feeling of commitment and the intrinsic rewards associated with challenging, novel, and self-directed work [61,62,63]. Therefore, when individuals are committed to organizational goals and have high levels of identification, willingness, and affective attachment toward their leaders, then creative ideas are more likely to be generated and successfully implemented [18]. In this vein, employees with more affective organizational commitment have greater intentions to engage in new tasks and improve working processes in the organization, which can enhance individual creativity and enable organizations to remain flexible in rapidly changing and competitive markets [64]. For example, Hou et al. [65] found a positive connection between organizational commitment and creativity based on a sample of 134 employees. Moreover, Shah, Nisar, Kashif ur, and Ijaz ur [66] reported the significantly positive relationships between transformational leadership, organizational commitment, and creative behavior. Integrating these findings, affective organizational commitment might be a critical mediator in the relationship between transformational leadership and creativity. We thus propose the following hypothesis:

**Hypothesis** **5.** *Affective organizational commitment mediates the effect of transformational leadership on employee creativity.*

Transformational leaders can also influence the performance of employees by providing assistance, opportunities, and encouragement to increase their emotional attachment at work [23]. Consequently, the positive effect of affective organizational commitment on the effectiveness of work performance can be realized [67]. In other words, leaders with a high level of transformational leadership can promote their subordinates’ performance by enhancing their affective commitment to the organization. Especially in the hospitality sector, these committed employees provide the guarantees for high work performance and hotel profitability [68,69]. For instance, Shaw et al. [70] found that affective organizational commitment is a significant predictor of helpful behaviors and overall performance based on a sample of 226 employees at two companies. Meyer et al. [71] and Riketta [72] also reported that employee affective commitment to the organization has a strong positive relationship with their performance in the organization based on meta-analyses of earlier works. In this vein, a transformational leader might enhance followers’ affective attachment by supporting their needs, maintaining their desire to remain in the organization by promoting intellectual fulfillment, and increasing their feelings of belonging by raising their motivation; all these efforts contribute to the development of employee performance at work [19,54,70,73]. Consequently, this study proposes the following hypothesis:

**Hypothesis** **6.** *Affective organizational commitment mediates the effect of transformational leadership on employee performance.*

By contrast, Eisenberger et al. [15] and Settoon et al. [74] proposed that if employees feel organizations care about them and value their efforts, the resulting high levels of perceived organizational support can help enhance employee feelings of obligation and affective attachment to the organization. This mechanism appears to have positive influences with regard to employee affective organizational commitment, and employees with high perceptions of organizational support are more willing to work to improve the organization. In the competitive business environment of the hospitality industry, hotel employees’ affective attachment and feelings of obligation ultimately create value for their hotels, and the positive relationship between perceived organizational support and affective organizational commitment is well documented [75,76]. For example, Wayne et al. [20] indicated that employees’ perception of organizational support is positively related to affective organizational commitment and organizational citizenship behavior, based on a follower–leader dyadic sample of 211 employees. Gakovic and Tetrick [77] reported that higher levels of perceived organizational support and stronger exchange relationships with organizations can increase employees’ feelings of obligation to the organization and increase organizational commitment. Based on a sample of 225 workers, Maertz et al. [78] also found that employees’ perceptions of organizational support have significant influences on employees’ affective organizational commitment and turnover behavior.

In addition, given the labor-intensive nature of the hotel industry, hotels are required to retain several creative employees to maintain competitive advantages in this dynamic business environment [4,47,48]. Therefore, Eisenberger et al. [15] and Amabile et al. [18] argued that individuals are more willing to engage in creating novel ideas and undertaking new tasks if they feel well supported and cared for by their organizations. For instance, based on a five-year study, Fitch [79] proposed that organizations that value employees with mutual trust and open communication can enhance their commitment and free up their creative development. Based on 2-wave time-lagged data from 1,059 employees, Chang, Jia, Takeuchi, and Cai [80] also found that organizations emphasize that high commitment to work can nourish individual creativity. In this vein, those committed to their organizations with a greater affective attachment can have better creative outcomes than those who are not [48,65]. On the basis of prior studies and the various theoretical arguments set out in them, we propose the following hypothesis:

**Hypothesis** **7.** *Affective organizational commitment mediates the effect of perceived organizational support on employee creativity.*

According to Eisenberger et al. [16], perceived organizational support helps employees and organizations build reciprocal relationships through mutual trust. Moreover, these reciprocation processes strengthen employees’ affective commitment and promote job performance. In other words, if employees believe their organizations respect their contributions and pay attention to their well-being, high performance will be achieved with these committed employees [52]. Therefore, perceived organizational support could positively influence work performance through affective organizational commitment. In line with this proposition, earlier studies revealed that employees’ affective organizational commitment plays a mediating role linking perceived organizational support and employee performance. For example, Choi [81] indicated the existence of positive relationships between perceived organizational support, affective organizational commitment, and job performance based on large-scale longitudinal data. Su, Baird, and Blair [82] also showed that organizational factors, such as perceived organizational support and job satisfaction, can significantly influence employee organizational commitment and ultimately enhance work performance. Similarly, Kuvaas [49] found that employees with high perceived organizational support and affective organizational commitment strengthen the development of work performance. Accordingly, this study proposes the following hypothesis:

**Hypothesis** **8.** *Affective organizational commitment mediates the effect of perceived organizational support on employee performance.*

## 3. Methods

### 3.1. Participants and Procedures

Prior to the process of data collection, back translation was performed to ensure the quality of the translation of the questionnaire [83]. We invited one bilingual professor to help translate all the items in the questionnaire from English to Chinese, and these items were then translated back to English with the help of another bilingual professor. Human resources (HR) managers were contacted by the authors to seek their assistance in our study, and we then collected data from 18 international tourist hotels in North Taiwan. With the help of the HR department, the respondents were drawn from all functional areas of the organization and included frontline employees and back office employees. We also visited these hotels several times before designing the questionnaire and met with management teams to discuss the job descriptions used at the hotel. This process helped the resulting instrument to better gather the genuine attitudes and behaviors of employees. In addition, the company’s internal system was used to distribute the questionnaires to potential respondents, who were instructed to complete the survey and return it using the attached envelope, with their confidentiality guaranteed. To lower the potential effects of common method variance (CMV), in which variances are influenced by the measurement method, instead of representing the correct meanings of constructs [84], the data were collected from multiple sources, including both the leaders and their subordinates. Each supervisor rated their subordinates’ creativity and performance at work, while each employee completed a questionnaire about his or her perceptions of transformational leadership, perceived organizational support, and affective organizational commitment. In this study, each supervisor was provided with a set of questionnaires: one questionnaire for the leader and another for the multiple employees who directly report to this supervisor. The survey process was anonymous; nonetheless, to ensure the data collection of multisource supervisor–subordinate data, marked numerical codes were used to match the responses from subordinates with their coordinating supervisors. Each completed questionnaire was separately returned in prepaid envelopes, and participants received a USD 3.0 voucher of appreciation. We distributed 800 questionnaires and excluded missing data from 12 employees with no leader response, 8 supervisors with no subordinate response, and 15 incomplete questionnaires. Accordingly, 362 completed and acceptable questionnaires were used in our study. The employees’ average age was 35.87 (ranging from 25 to 65), and the employees’ average company tenure was 5.63 years (ranging from 1 to 25 years); additionally, men and women accounted for 172 (47.51%) and 190 (52.49%) in our sample, respectively. Most of the employees had received a college or university education (82%), with only 5.1% of employees receiving only high school diplomas.

### 3.2. Measures

***Transformational leadership***. Transformational leadership was measured with 20 items from the Multifactor Leadership Questionnaire (MLQ) Form 5X-Short [85]. Subordinates indicated their degree of disagreement or agreement with a number of statements using a five-point Likert scale, ranging from “strongly disagree” (1) to “strongly agree” (5). Sample items for the four components in the survey are as follows: intellectual stimulation (e.g., “My leader looks for different points of view when they solve problems”), idealized influence (e.g., “My leader talks to us about the importance of values and ethics in the organization”), individualized consideration (e.g., “My leader spends time on coaching and teaching me”), and inspirational motivation (e.g., “My leader stresses the importance of having a general sense of achieving our missions”). The overall Cronbach’s alpha was equal to 0.93 (alpha > 0.70), indicating satisfactory reliability.

***Perceived organizational support.*** We used the short version of the Survey of Perceived Organizational Support [15] to measure employees’ perceptions of organizational support with four items. A seven-point Likert scale was used, ranging from “strongly disagree” (1) to “strongly agree” (7), to gather information regarding employee perceived organizational support. Sample items are, “My organization values my contributions,” and “My organization provides help when I have a problem.” The Cronbach’s alpha was equal to 0.81 (alpha > 0.70), demonstrating good internal consistency and reliability.

***Affective organizational commitment***. Affective organizational commitment was assessed using Allen and Meyer’s three-component model [23] with eight items. Employees used a seven-point Likert scale, ranging from “strongly disagree” (1) to “strongly agree” (7), to reflect their organizational commitment status. Sample items, are “I would be very happy to spend the rest of my career in this organization,” and “This organization has a great deal of personal meaning for me.” The overall Cronbach’s alpha was equal to 0.90 (alpha > 0.70), suggesting good reliability.

***Creativity.*** We measured individual creativity with thirteen items developed and validated by Zhou and George [27]. Leaders rated each employee’s creativity with a five-point Likert scale, ranging from “strongly disagree” (1) to “strongly agree” (5). Sample items are, “This employee is a good source of creative ideas,” and “This employee always suggests new ways to achieve objectives and goals.” The Cronbach’s alpha was equal to 0.92 (alpha > 0.70), demonstrating good reliability.

***Performance.*** We followed Janssen and Van Yperen’s [86] suggestion and used five items from Podsakoff and MacKenzie [87] to measure employees’ job performance. Leaders rated each subordinate’s performance using a seven-point Likert scale, ranging from “strongly disagree” (1) to “strongly agree” (7). Sample items are “This employee meets all the formal performance requirements of his/her job,” and “This employee always completes the duties in the job description.” The Cronbach’s alpha was equal to 0.84 (alpha > 0.70), showing acceptable internal consistency and reliability.

### 3.3. Analysis Strategy

The two-step strategy presented in Anderson and Gerbing [88] was used to investigate the proposed model. First, confirmatory factor analysis (CFA) was used in the measurement model to examine the fit of the overall model, and structural equation modeling (SEM) analyses were then performed to test the structural model according to the results of this process in Table 1. We examined the overall model fit using maximum-likelihood estimation with the aid of AMOS 17.0 [89]. In addition, the fit indices of the chi-square (χ^2^) value, degrees of freedom (df), χ^2^/df value, comparative fix index (CFI), goodness-of-fit index (GFI), adjusted goodness-of-fit index (AGFI), Bollen’s incremental fit index (IFI), Bentler–Bonett normed fit index (NFI), standardized root mean square residual (SRMR), and the root mean square error of approximation (RMSEA) were all used to test the overall model fit [90,91,92,93].

## 4. Results

As mentioned previously, CFA analyses were used to test the measurement model, and the results revealed a good fit to the data (χ^2^ = 2482.4, df = 1070, χ^2^/df = 2.32, GFI = 0.95, AGFI = 0.95, NFI = 0.95, IFI = 0.95, CFI = 0.96, SRMR = 0.03, and RMSEA = 0.04). Although we faced the limitations of the chi-square likelihood ratio (*p* < 0.001) with a larger sample size, these results can be expected, as our observed variables were greater than 30 [94]. We then assessed the normality on the basis of the skewness and kurtosis values. The results of the skewness ranged from −2.43 to 0.29, with values less than 3.0, while the kurtosis ranged from 1.54 to 8.93, with values less than 10; thus, the distributional normality was acceptable [95]. In addition, the values of the residuals ranged from 0.07 to −0.08 in the covariance matrix, while those in the standardized residual covariance matrix were 5 times greater than 2.0 (up to 3.64). We reported that the standardized residuals are sensitive to sample size; thus, a small difference in the covariance residuals may produce significant standardized residuals [92,93].

Moreover, the results of the composite reliability (CR) ranged from 0.87 to 0.96, over the 0.60 CR threshold value, thus providing evidence of internal consistency reliability [96,97]. Meanwhile, the factor loadings of each items in the five-factor model were all significant (all *p* < 0.001), and the average variance extracted (AVE) ranged from 0.60 to 0.62, over the 0.50 AVE threshold value [96,97]; thus, the convergent validity was supported. Most important of all, compared with the one-factor model (all items loaded on the same factor), three-factor models (i.e., transformational leadership, perceived organizational support, and creativity loaded on the same factor—affective organizational commitment and performance), and four-factor models (i.e., transformational leadership and creativity loaded on the same factor—affective organizational commitment, perceived organizational support, and performance), the proposed five-factor model shown in Table 2 showed a significantly better fit with the data, based on the results of the chi-squared difference tests. Consequently, the CFA results offer strong support for the validity of the five-factor model while providing evidence that common method variance did not seriously influence the estimation results [84].

**Figure 2:**
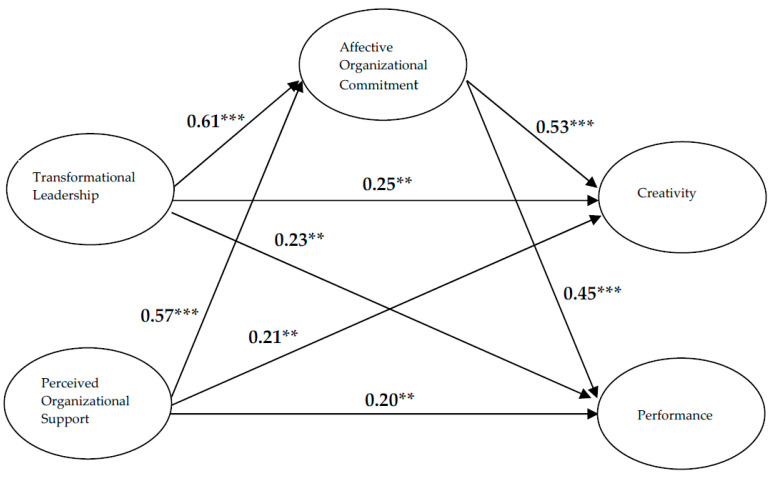
Structural equation modeling of the proposed model. Note: (1) N = 362, ** *p* < 0.01; *** *p* < 0.001 (two-tailed). (2) χ^2^ = 2111.72, df = 806, χ^2^/df = 2.62, GFI = 0.95, AGFI = 0.95, NFI = 0.95, IFI = 0.95, CFI = 0.95, SRMR = 0.03, and RMSEA = 0.04.

Table 3 provides the means, standard deviations, and correlations for the variables used in this study. We then examined the structural model, and the results also demonstrated a good fit to the data (χ^2^ = 2,111.72, df = 806, χ^2^/df = 2.62, GFI = 0.95, AGFI = 0.95, NFI = 0.95, IFI = 0.95, CFI = 0.95, SRMR = 0.03, and RMSEA = 0.04). In addition, Hypothesis 1 proposes that transformational leadership is positively related to employee creativity. Figure 2 shows that the result for the direct relationship between transformational leadership and employee creativity is significant and positive (standardized direct effect = 0.25, *p* < 0.01). Consequently, Hypothesis 1 is supported. Moreover, as predicted in Hypotheses 2 through 4, transformational leadership is positively linked with employee performance (standardized direct effect = 0.23, *p* < 0.01), perceived organizational support is positively associated with employee creativity (standardized direct effect = 0.21, *p* < 0.01), and perceived organizational support is positively linked with employee performance (standardized direct effect = 0.20, *p* < 0.01). Therefore, Hypotheses 2, 3, and 4 are also supported.

To investigate Hypotheses 5 through 8, to determine whether employee affective organizational commitment mediates the relationships of transformational leadership and perceived organizational support with employee creativity and performance, we examined the conditions of mediation. We followed Baron and Kenny’s [98] causal steps strategy stating that the independent variable must influence the mediating and dependent variables, while the mediating variable must influence the dependent variable. Figure 2 shows that transformational leadership has a significant and positive relationship with employee affective organizational commitment (standardized direct effect = 0.61, *p* < 0.001), perceived organizational support has a significant and positive relationship with employee affective organizational commitment (standardized direct effect = 0.57, *p* < 0.001), employee affective organizational commitment has a significant and positive relationship with employee creativity (standardized direct effect = 0.53, *p* < 0.001), and employee affective organizational commitment has a significant and positive relationship with employee performance (standardized direct effect = 0.45, *p* < 0.001). Therefore, the conditions of mediation are supported.

Finally, we adopted the Sobel [99] test to examine our results. As shown in Table 4, the Sobel test results provide evidence for the positive and significant indirect effect of affective organizational commitment on the relationship between transformational leadership and employee creativity (standardized indirect effect = 0.32, Z = 6.07, *p* < 0.01), the relationship between transformational leadership and employee performance (standardized indirect effect = 0.27, Z = 5.43, *p* < 0.01), the relationship between perceived organizational support and employee creativity (standardized indirect effect = 0.30, Z = 5.92, *p* < 0.01), and the relationship between perceived organizational support and employee performance (standardized indirect effect = 0.26, Z = 5.32, *p* < 0.01). As a result, Hypotheses 5, 6, 7 and 8 are all supported.

## 5. Discussion

### 5.1. Theoretical Implications

This study was undertaken in an effort to synthesize leadership, social exchange, and creativity theories in the context of hospitality by examining the hypothetical and empirical links among transformational leadership, perceived organizational support, affective organizational commitment, creativity, and performance. Previous studies of both transformational leadership and employee perceived organizational support found direct links between employee affective organizational commitment, creativity, and performance [9,16,20,31]. This study proposed that affective organizational commitment is a critical mediator linking transformational leadership, perceived organizational support, creativity, and performance. Most important of all, this study extends the findings of previous works, which were mostly carried out in Western contexts, as it was conducted on the basis of data from Taiwanese employees. The ways in which the empirical conclusions of this work extend the findings of previous studies are discussed below.

First, previous studies have separately revealed the positive relationships of transformational leadership with employee creative behavior and work performance, and we thus contribute new knowledge regarding the theory by simultaneously integrating these relationships and examining them in a corporate setting. The results of this work show that transformational leaders can improve employees’ intrinsic motivation by affecting their basic values, beliefs, and attitudes, which all contribute to the achievement of organizational goals and the enhancement of individual creativity and performance [7,8,9,10]. Given that creativity has been widely recognized as an important ingredient for organizational effectiveness [18,30], these highly motivated employees influenced by transformational leaders tend to obtain and apply novel knowledge and skills needed at their work, as well as achieve better job performance [8,36]. Relative to the hospitality sector during the COVID-19 pandemic period, with these positive influences from transformational leadership, hotel employees can thus provide better knowledge-intensive services with greater creativity and higher performance [1,2,4,37,38].

The second conclusion of this study extends the findings of the previous works on the consequences of perceived organizational support. As discussed above, Eisenberger et al. [15] defined perceived organizational support as employees’ perception that their organizations care about them and value their contributions, and such support is positively linked with enhanced employee work attitudes and behaviors. Our empirical results reveal that organizations can demonstrate their support for employee creativity and performance by using encouragement, reward, respect, and other forms of recognition. That is, if employees perceive that they are valued by their organizations, they are more likely to successfully implement novel ideas and exhibit improved work performance [27]. Given the labor-intensive nature of the hotel business, employees’ perceived organizational support is a key source of their enhanced creativity and performance at work, encouraging them to provide better processes, services, and strategies for their hotels [37].

Our third conclusion extends the leadership and social exchange theory by investigating employee affective organizational commitment as a mediator of the influences of transformational leadership and perceived organizational support on creativity and performance. More specifically, this study is the first to examine the mediating role of affective organizational commitment using SEM bootstrapping analyses to provide a clear picture linking these mechanisms. The results of this work reveal that employee affective organizational commitment is a critical mediator among these relationships because affective organizational commitment reflects the psychological connection between employees and their organization; such linkage encourages them to voluntarily stay in the organization and make greater contributions to achieving its aims [100]. In other words, given that transformational leadership is a contextual factor that affects employees’ attitudes and behaviors at work, the results of this study show that transformational leaders can enhance subordinates’ affective organizational commitment and their feelings of belonging to the organization by showing consideration, providing support, and serving as role models, thus improving their ability to generate novel ideas and achieve higher levels of performance [9,31]. We also prove that organizations can influence the creative behavior and performance of employees by providing assistance, opportunities, and encouragement to increase their emotional attachment at work [15,23]. Most important of all, employees with high affective organizational commitment likely remain in their organizations for longer and thus contribute greater efforts to achieving their tasks and improving existing work processes, enabling organizations to remain flexible in rapidly changing business markets [64]. Our third conclusion thus extends the finding of previous studies that transformational leadership and perceived organizational support can both enhance employees’ affective attachment, thus improving their creativity and performance at work. Especially in the competitive hospitality business environment of the post COVID-19 era, employees with high commitment to their hotels can thus contribute to providing better services, ultimately promoting hotel profitability [101,102].

### 5.2. Managerial Implications

Overall, the findings of this study suggest that leaders with high transformational behaviors and employees with more perceived organizational support can both enhance the psychological links among subordinates’ affective organizational commitment, creative behavior, and performance. We thus propose that companies in the hospitality industry should work to create a friendly environment to promote personal and social interactions between organizations and employees, as well as leaders and subordinates. Human resource departments in hotel companies can also use suggestions from Bass [10] in the description of transformational leadership factors, namely, charisma (the imposition of the direction of action and vision, triggering the enthusiasm of others; pride, confidence in one’s own strength through the power of personal attractiveness, and emotional appeal) and individual attention (focusing on the needs of subordinates and especially giving special rewards and organizing ceremonies to celebrate excellence and superior results) to provide effective training programs for employees and their leaders to increase the quality of supervisor–subordinate dyadic relationships. Such measures not only create a positive atmosphere between companies, leaders, and employees, but ultimately help reduce employee turnover intention and enhance their motivation to provide better customer services. Most important of all, given that employees are the most valuable assets of hotels and that most of them have a direct or indirect opportunity to serve customers, management systems and leaders in hotels can show extra allowance, welcome, and acceptance of employees’ efforts to determine better processes and services in hotel operations. This consideration and support for employees can thus enhance their affective attachment in hotels and eventually generate high creativity and performance at work. Therefore, the sustainable competitive advantage can be achieved with these talented and motivated employees who are recognized and rewarded for their creativity and performance [8,18].

### 5.3. Limitations

In considering the findings and implications of this study, a number of limitations should be recognized. First of all, although our findings are consistent with those of previous studies and the theoretical predictions, we can make inferences, but we cannot confidently determine the causality among these variables due to the cross-sectional design of this work. Another limitation of this work is that the use of self-reported data can potentially be influenced by common method variance [84]. However, the data on employee creativity and performance were obtained from supervisors’ ratings and are thus relatively unbiased. The CFA results also showed that the proposed five-factor model had better fit to the data than the other models examined in this work, based on the chi-squared difference tests, all of which help to reduce concerns about common method variance. Finally, this work did not attempt to seek empirical evidence to support whether or not supervisors with different leadership styles are also able to promote employees’ affective organizational commitment and thus enhance their creativity and performance.

### 5.4. Future Research Direction

Although the comprehensive research design and evidence-based results yield insights in this study, some suggestions are listed for future research directions. First, for researchers to investigate dynamic instead of static views, future studies can track changes in transformational leadership and perceived organizational support over time to strengthen the results of this study by carrying out longitudinal research in organizational settings. Second, to stay ahead of the competitive business competition and provide a broader research perspective with fewer common method variance concerns, future studies should make use of proxies, such as big data obtained from organizational databases over a period of time, to better determine employee creativity and performance. Finally, to better understand how a person behaves while leading a group and the emotional attachment of those who work for him/her, future studies could explore the joint influences of other leadership styles on employee affective organizational commitment, along with transformational leadership, and work to clarify this issue.

### 5.5. Conclusions

In conclusion, in the context of hospitality, our study provides further support for the view that transformational leadership and perceived organizational support are both related to employee affective organizational commitment, creativity, and performance, while affective organizational commitment is related to employee creativity and performance. This finding demonstrates that both transformational leaders and employees’ perception of organizational support in hotels can effectively enhance employee affective organizational commitment, creativity, and performance, while both transformational leaders and employees’ perceived organizational support can promote creativity and performance through affective organizational commitment.

## Figures and Tables

**Table 1:** Fit indices.

Fit Indices	Model Value	Reference Value	Overall Model Fit
χ^2^	2482.4		Acceptable
df	1070		
χ^2^/df	2.32	<5.00	Yes
CFI	0.96	>0.90	Yes
GFI	0.95	>0.90	Yes
AGFI	0.95	>0.90	Yes
IFI	0.95	>0.90	Yes
NFI	0.95	>0.90	Yes
Standardized RMR	0.03	<0.05	Yes
RMSEA	0.04	<0.05	Yes
RMSEA lower boundary of 90% confidence interval	0.04		
RMSEA upper boundary of 90% confidence interval	0.05		

**Table 2:** Confirmatory factor analysis results.

Model	χ^2^	df	Probability	χ^2^/df	Δχ^2^ (Δdf)	GFI	NFI	CFI	SRMR	RMSEA
*Measurement models*										
**5-Factor model**	**2482.40**	**1070**		**2.32**	**-**	**0.95**	**0.95**	**0.96**	**0.03**	**0.04**
4-Factor model	7198.67	1074	*p* < 0.001	6.83	4716 (4) ***	0.85	0.84	0.84	0.08	0.09
4-Factor model	6487.82	1074	*p* < 0.001	6.47	4005 (4) ***	0.86	0.86	0.86	0.07	0.12
3-Factor model	16,745.73	1077	*p* < 0.001	14.51	14,263 (7) ***	0.81	0.80	0.80	0.11	0.16
3-Factor model	16,841.54	1077	*p* < 0.001	14.35	14,359 (7) ***	0.80	0.80	0.80	0.11	0.17
1-Factor model	26,540.03	1080	*p* < 0.001	23.31	24,058 (10) ***	0.78	0.77	0.77	0.14	0.21

Note: N = 362, df = df for χ^2^, *p* = significance of χ^2^, *** *p* < 0.01.

**Table 3:** Means, standard deviations, and correlations for the variables.

	Mean	S.D.	1	2	3	4	5	6	7	8	9
1. Age	2.19	0.58	-								
2. Tenure	1.73	0.76	0.31 **	-							
3. Gender	1.56	0.43	−0.20 **	0.02	-						
4. Education	3.29	0.49	−0.13 **	−0.27 **	−0.19 **	-					
5. Transformational leadership	3.93	0.65	0.05	0.10 *	0.09 *	−0.07	**(0.79)**				
6. Perceived organizational support	5.87	0.72	0.15 **	0.10 *	0.10 *	0.02	0.68 **	**(0.79)**			
7. Affective organizational commitment	5.91	0.74	0.10 *	0.22 **	0.02	−0.10 *	0.71 **	0.62 **	**(0.79)**		
8. Creativity	3.84	0.53	−0.03	−0.04	0.03	0.14 **	0.52 **	0.33 **	0.55 **	**(0.77)**	
9. Performance	5.62	0.68	0.02	−0.15 **	−0.09 *	0.09 *	0.42 **	0.36 *	0.47 *	0.72 **	**(0.78)**

Note: (1) N = 362, * *p* < 0.05; ** *p* < 0.01 (two-tailed). (2) The square root values of AVE for discriminant validity are shown in parentheses along the diagonal.

**Table 4:** Sobel tests of the statistical significance of indirect effects.

Independent Variable	Mediator Variable	Dependent Variable	Standardized Indirect Effect	Z Value	Two Tailed Significance
Transformational leadership	Affective organizational commitment	Creativity	(0.61) × (0.53) = 0.32	6.07	**
Transformational leadership	Affective organizational commitment	Performance	(0.61) × (0.45) = 0.27	5.43	**
Perceived organizational support	Affective organizational commitment	Creativity	(0.57) × (0.53) = 0.30	5.92	**
Perceived organizational support	Affective organizational commitment	Performance	(0.57) × (0.45) = 0.26	5.32	**

Note: N = 362, ** *p* < 0.01 (two-tailed).

## Data Availability

The data included in this study are available from the author upon request.
